# Sim-SEA: Integrating Significant Event Audit and In Situ Simulation to Support Patient Safety Learning and Organizational Change—A Prospective Single-Center Pilot Study in Pediatric Emergency Care

**DOI:** 10.3390/healthcare14213810

**Published:** 2026-11-08

**Authors:** Davide Silvagni, Simona Spada, Vittoria Berveglieri, Francesco Corazza, Pierantonio Santuz, Roberto Benoni, Silvia Poli, Stefano Tardivo, Pier Luigi Ingrassia, Francesca Moretti

**Affiliations:** 1 Department of Mother and Child, Verona Integrated University Hospital Trust, Piazzale Aristide Stefani 1, 37126 Verona, Italy; davide.silvagni@aovr.veneto.it (D.S.); francesco.corazza@aovr.veneto.it (F.C.);; 2 National Center for Global Health, Italian National Institute of Health (Istituto Superiore di Sanità), Viale Regina Elena 299, 00161 Rome, Italy; roberto.benoni90@gmail.com; 3 Department of Neurosciences, Biomedicine and Movement Sciences, University of Verona, Str. Le Grazie 8, 37134 Verona, Italy; silvia.poli@univr.it; 4 Department of Diagnostics and Public Health, University of Verona, Str. Le Grazie 8, 37134 Verona, Italy; stefano.tardivo@univr.it; 5 Centro Cantonale di Simulazione (CCSim), Centro Professionale Sociosanitario Medico-Tecnico, Via Ronchetto 14, 6900 Lugano, Switzerland; pierluigi.ingrassia@gmail.com

**Keywords:** in situ simulation, significant event audit, clinical risk management, patient safety, translational simulation, organizational learning, pediatric emergency care, clinical governance

## Abstract

Background/Objectives: Patient safety depends on healthcare systems’ ability to learn from clinical experience and translate insights into system-level improvement. Significant Event Audit (SEA) is well established but may only partly capture the dynamic, context-dependent nature of clinical performance. Although in situ simulation is increasingly used to explore team performance and system vulnerabilities, its routine integration into formal risk-management pathways remains limited. We developed Sim-SEA, a structured approach embedding in situ simulation within the SEA learning cycle. Methods: Sim-SEA adapted the seven-phase SEA framework to incorporate in situ simulation as a core component. We conducted a 12-month single-center, uncontrolled pre–post pilot study in a tertiary pediatric emergency department, comprising 10 Sim-SEA cycles. Self-perceived competence in technical and non-technical domains was assessed at baseline and immediately after simulation using a 6-item ad hoc questionnaire. A follow-up assessment was conducted among participants who subsequently encountered a real emergency. Participants’ perceptions, system vulnerabilities, and improvement actions were also examined. Results: Fifty-nine physicians, residents, and nurses participated. Self-perceived competence improved significantly from baseline to post-simulation across ABCDE assessment, teamwork, situational awareness, communication, speak-up behavior, and decision-making (all *p* < 0.01). At follow-up, available for 35/59 participants (59.3%) who subsequently encountered a real emergency, self-perceived scores remained significantly higher than baseline. Implemented changes included workspace reorganization, a cognitive medication aid, and a standard operating procedure for role allocation. Participants reported greater engagement, reflective learning, psychological safety, and awareness of risks and system vulnerabilities. Conclusions: In this single-center, uncontrolled pilot study, Sim-SEA was feasible as a structured method for embedding in situ simulation within clinical risk management and supported potential benefits for self-perceived learning and the identification and development of organizational and environmental improvement actions. Further studies should assess effects on objective competence and patient-safety outcomes.

## 1. Introduction

Patient safety relies on healthcare systems’ ability to function as learning organizations, continuously translating clinical experience into system-level improvement [1]. Incident reporting (IR) systems and retrospective analysis tools, such as the Significant Event Audit (SEA), have traditionally represented a central component of patient safety programs [2,3].

Although these approaches can support collective reflection and the identification of contributory factors, they have important limitations when applied within complex healthcare environments [3]. As they rely on reconstructed accounts of events, they may only partially capture the dynamic and context-dependent nature of real-world performance and team interactions and remain vulnerable to cognitive biases, such as hindsight and recall bias [4,5]. Moreover, insights generated through retrospective analysis may not consistently translate into sustained system-level change because of limited feedback mechanisms, inconsistent implementation of corrective actions, and insufficient evaluation of their effectiveness [6,7].

At the same time, there is increasing recognition of the need to complement a Safety-I perspective—focused on learning from failure—with a Safety-II approach that emphasizes understanding how everyday performance succeeds through adaptation and the capacity of systems to maintain resilience [8,9]. From this perspective, safety emerges through real-time interactions, non-technical skills, and teams’ responses to evolving clinical conditions. Although SEA provides a structured method for reflecting on significant events, on its own it offers limited access to the adaptive, relational, and contextual dimensions of practice which are critical for understanding how teams coordinate, communicate, and respond to complexity in real time [10].

In situ simulation offers a complementary method for examining clinical work as it unfolds, enabling teams to explore performance, workflows, environmental conditions, and system vulnerabilities within the actual care context [11,12]. Although in situ simulation is increasingly used to explore care processes, its routine integration into formal clinical risk-management and governance pathways remains limited. In particular, it is not always linked to the analysis of specific significant events or embedded within a structured cycle of improvement and follow-up. These considerations are particularly relevant in pediatric emergency care, where low-frequency, high-impact events may unfold rapidly and under considerable time pressure [13]. Age-related physiological variability, sudden clinical deterioration, and unpredictable changes in patients’ conditions require continuous adaptation and tightly coordinated team responses. Opportunities for experiential learning from these events are limited, and retrospective approaches alone may not adequately support future preparedness [14].

To operationalize this integration within an established audit pathway, we developed Sim-SEA, a structured approach that embeds in situ simulation within the SEA learning cycle. Sim-SEA links retrospective analysis of real clinical events with context-based system exploration, experiential team learning, implementation of improvement actions, and iterative reassessment [11,12]. This integration extends traditional audit processes by linking retrospective reflection with prospective, experiential learning and system testing. This approach aligns with recent recommendations advocating for the integration of simulation into clinical governance and patient safety processes, emphasizing context-sensitive, sustainable, and high-impact strategies [1].

The primary aims of this pilot study were:

- To describe the development of Sim-SEA.
- To describe the implementation of Sim-SEA within the clinical risk-management pathway of a pediatric emergency department and explore healthcare professionals’ perceptions of the approach.

A secondary, exploratory aim was

- To report preliminary findings related to self-perceived technical and non-technical competence and to document system vulnerabilities and organizational and environmental improvement actions arising from its implementation in the shock room of a pediatric emergency department.

## 2. Materials and Methods

This prospective single-center uncontrolled pre–post pilot study evaluated the implementation and preliminary findings of the Sim-SEA over a 12-month period, from January to December 2024. Sim-SEA was implemented as a quality improvement initiative; the study was reported in accordance with the SQUIRE 2.0 guidelines for quality improvement studies [15]. The simulation-specific aspects of the manuscript were additionally informed by the Reporting Guidelines for Health Care Simulation Research [16]. A complete SQUIRE-2.0 checklist is provided in Supplementary File S1 The project was initially conducted as a routine educational and quality improvement activity under institutional administrative authorization. Although the manuscript was submitted prior to formal ethical approval, approval was subsequently granted by the Institutional Review Board of the University of Verona (CARP) on 12 October 2026 (Approval No. 2026-14).

### 2.1. Participants and Setting

This study was conducted in a tertiary Pediatric Emergency Room (PER) of a University Hospital in Northern Italy, serving as a referral hub for pediatric emergencies within the regional network. The unit manages approximately 20,000 admissions per year and includes a 10-bed intensive short-stay observation area. All frontline healthcare professionals involved in emergency care—including senior physicians, nurses, and residents rotating through the pediatric emergency department during the study period—were eligible and invited to participate in the Sim-SEA program through structured departmental meetings and internal staff communication.

### 2.2. The Sim-SEA Approach

SEA is a structured quality improvement method based on team reflection and collective analysis of clinically relevant events [17]. It supports learning from a wide range of situations, including adverse events, near misses, complex cases, and instances of successful care.

By integrating SEA with in-situ simulation, Sim-SEA extends this reflective process into an experiential and context-sensitive exploration of the event trajectory. Recreating the event within the real clinical environment allows clinical teams to revisit the complexity and dynamic context in which care was delivered to examine not only what happened, but also how team behaviors, environmental conditions, workflows, resources, and organizational constraints shaped performance. This process facilitates the identification of latent safety threats and modifiable system factors, while also supporting team learning and the development of non-technical skills. The Sim-SEA process followed a structured sequence of phases adapted from the traditional SEA framework, with in situ simulation embedded as a core component of the learning cycle, from event identification to the implementation and review of improvement actions. The main phases and components are summarized in Table 1, while a comparison between the traditional SEA framework and its Sim-SEA adaptation is provided in Supplementary File S2. For each selected event, scenarios were developed from the analysis of real clinical cases to recreate the key clinical, contextual, and relational dynamics of practice.

### 2.3. Implementation of the Sim-SEA Approach: Pilot Study Design and Iterative Cycles

**Figure 1:**
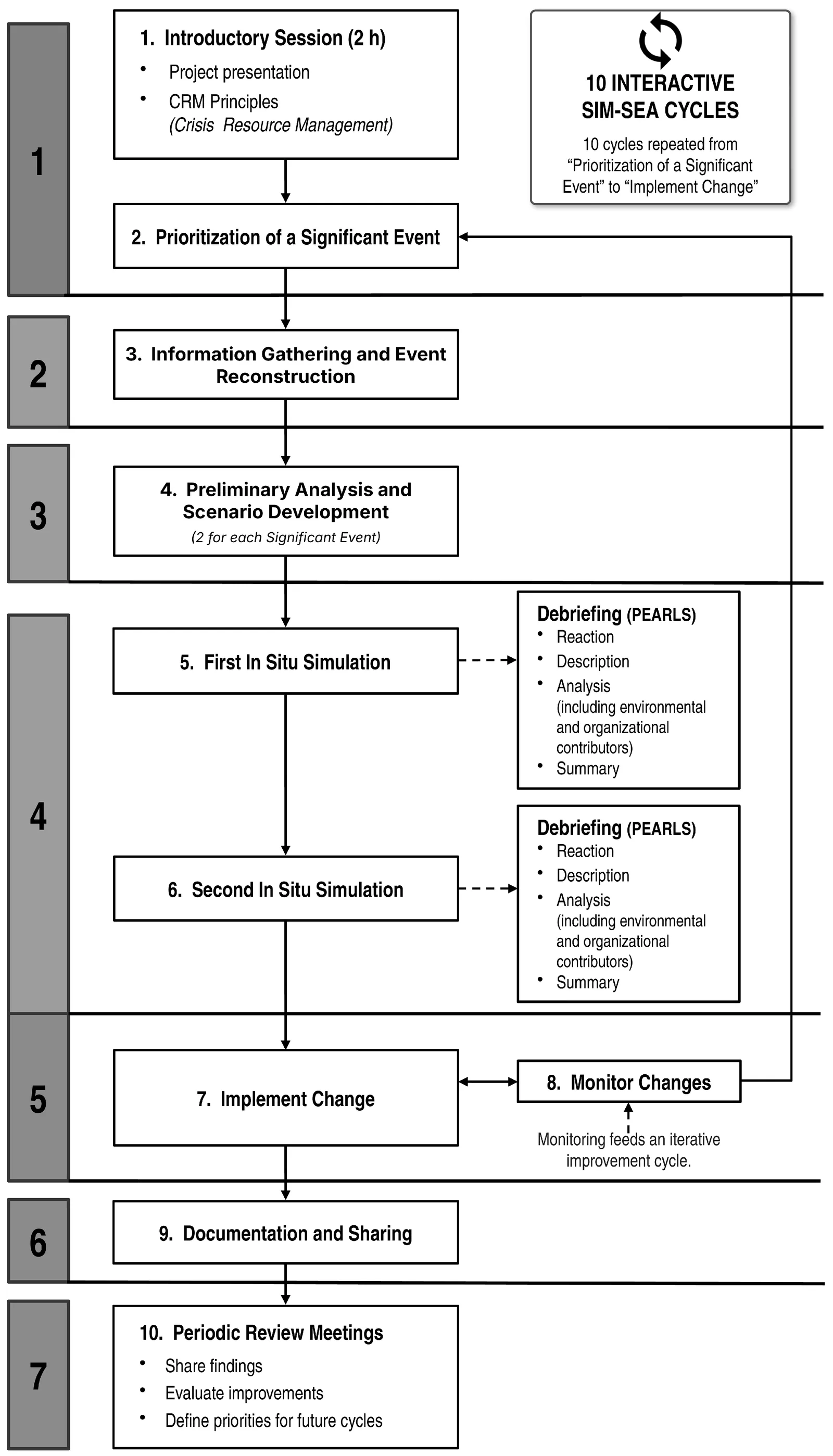
Project design.

The Sim-SEA approach was implemented as an iterative process, structured into a series of cycles. Figure 1 provides an overview of the overall design.

Before the first Sim-SEA cycle, participants attended a 2-h introductory session covering the rationale and procedures of the program and providing a brief overview of core Crisis Resource Management (CRM) principles relevant to the simulations. Participants received preparatory reading materials in advance to support shared foundational understanding and optimize engagement.

Each Sim-SEA cycle comprised two in situ simulation sessions informed by the learning and system-improvement priorities identified through the analysis of the selected significant event. Each simulation lasted approximately 15 min and was followed by a 45-min structured debriefing. In each session, the team consisted of one physician acting as team leader, two nurses, and three residents, reflecting the usual composition of the shock-room team during high-acuity pediatric emergencies.

Participation was organized on a rotational basis to ensure involvement of all healthcare professionals (nurses, residents, and physicians). Each participant attended one Sim-SEA cycle only.

Following completion of the 10 Sim-SEA cycles, a final meeting involving all participants was held, in accordance with Phase 7 of the Sim-SEA framework, to review the findings and discuss the changes implemented and the overall impact of the process.

### 2.4. Significant Event Selection, Analysis and Scenario Development

Significant events were identified through the existing institutional incident-reporting system. Following consultation with the staff involved, identified events were considered for potential inclusion in a Sim-SEA cycle.

A multidisciplinary Core Project Team (CPT) comprising three simulation experts, one risk-management expert, the nurse coordinator of the Clinical Unit, and three representatives of frontline staff reviewed the significant events identified during Phase 1. Events were prioritized according to predefined criteria, including clinical relevance, frequency, severity, relevance to current practice, and potential to identify organizational or system-level improvement opportunities, including implications for team performance.

Incident reports were de-identified in accordance with institutional procedures.

The analysis of each significant event followed Phase 3 of the Sim-SEA framework. Consistent with the aims of SEA the analysis was intended to support collective learning and identify opportunities for system improvement. The Five Whys technique was used as a pragmatic questioning tool to examine both factors contributing to risk and protective or adaptive factors that supported successful performance from a human factor’s perspective [19,20].

Throughout the process, the focus remained on the event trajectory, system conditions, and contributing factors rather than on individual staff members, consistent with the non-blaming and psychologically safe approach adopted for routine institutional incident reporting and analysis. The human-factor perspective was operationalized by focusing on how performance was shaped by the interaction among people, team dynamics, task demands, workflows, equipment, the physical environment, and organizational conditions. The Yorkshire Contributory Factors Framework informed this multidimensional analysis [21]. Particular attention was paid to critical decision points and moments of adaptation, examining how interactions among these factors either increased risk or supported an effective response. The findings were translated into technical and non-technical learning needs at the individual and team levels, together with system-improvement priorities. These informed the development of event-based simulation scenarios designed to recreate the key clinical, cognitive, relational, environmental, and organizational conditions identified during the analysis. Scenarios were developed using the de-identified clinical information available from the event analysis. An example of this process is provided in Supplementary File S3.

### 2.5. Debriefing: The PEARLS Framework

Debriefing was conducted using the PEARLS framework (Promoting Excellence and Reflective Learning in Simulation), following its four phases—reactions, description, analysis, and summary—to support structured reflection, shared sensemaking of events and team learning [22]. Debriefings were consistently facilitated by the same group of three trained and experienced simulation educators. Facilitators supported participants’ self-assessment using a plus/delta approach (e.g., what went well and what could be improved), explored the reasoning and decision-making processes underlying their actions, and provided focused feedback when appropriate, while maintaining a psychologically safe and non-judgmental learning environment [23].

Given the dual aim of Sim-SEA—to support experiential team learning while also exploring system vulnerabilities—particular attention was directed to how clinical work unfolded within the real context of care. Environmental and organizational factors—such as workspace layout, equipment positioning, workflow, and resource availability— were explicitly explored to understand how they shaped team performance and to support system-level learning.

Each debriefing concluded with a summary phase aimed at consolidating learning and translating insights into action, including positive practices to be reinforced and concrete improvement actions or organizational changes.

To support collective sensemaking and the development of shared organizational memory, a whiteboard was used as a cognitive aid to capture key discussion points, agreed actions, and system-level recommendations. Photographs of the whiteboard were archived and shared with staff members who had not participated in the sessions, thereby extending learning beyond the simulation team and supporting broader organizational learning.

### 2.6. Evaluation and Data Collection

To explore participants’ perceptions and the perceived educational and practice-related impact of the Sim-SEA approach, a set of ad hoc questionnaires were administered at different time points.

Self-perceived competence in performing the patient first assessment using the ABCDE approach (Airway, Breathing, Circulation, Disability, and Exposure), as well as in core CRM domains, was assessed using a 6-item self-administered questionnaire rated on a 5-point Likert scale (Supplementary File S4). The items were developed by the study team, including experts in crisis resource management (CRM) and simulation, and informed by relevant literature. They were adapted to the objectives and clinical context of the Sim-SEA program. The non-technical skills considered relevant to shock room management included teamwork (role distribution, workload allocation, and shared goals), situational awareness (awareness of the clinical situation and its evolution, and effective use of available resources), communication (clear and structured information exchange, including closed-loop communication), speak-up behavior (requesting help, clarifying doubts, providing updates, and contributing relevant information), and decision-making (avoiding fixation errors, gathering and comparing information, and using cross-checks). Before its broader implementation, the questionnaire was reviewed during the first Sim-SEA cycle for item clarity and comprehensibility. Participants were invited to report any unclear or problematic wording; no comments requiring modification of the items were raised.

Assessments were conducted at baseline (T1) and immediately after completion of each Sim-SEA cycle (T2), following the second simulation session and its associated debriefing. At follow-up, at the end of all 10 Sim-SEA cycles (T3), the same self-perceived competence questionnaire was administered at follow-up to all available participants who had managed at least one real emergency in the shock room since their most recent simulation session. The assessment also included two additional ad hoc questions assessing the perceived usefulness of the Sim-SEA program in managing real emergencies and its perceived contribution to stress management during critical events.

At T3, a second ad hoc questionnaire was administered to all participants to assess overall satisfaction with the Sim-SEA approach and participants’ perceptions of the effectiveness of the organizational and environmental changes implemented through the Sim-SEA process. Finally, an open-ended question explored participants’ perceptions of the advantages and disadvantages of Sim-SEA compared with a conventional SEA approach.

### 2.7. Statistical Analysis

Descriptive statistics were used to summarize the data, including percentages and frequencies for categorical variables and medians with interquartile ranges (IQR) for continuous variables. Differences between subgroups (physicians, residents, nurses) at baseline were assessed through chi-squared and Fisher exact tests or the Kruskal–Wallis test, as appropriate.

Linear mixed-effects models (LMER) for longitudinal data were used to estimate changes over time in the six self-perceived competence outcomes: ABCDE assessment, teamwork, situational awareness, communication, speak-up behavior, and decision-making.

Random effects accounted for individuals nested within simulation groups by including separate random intercepts for simulation groups and individuals, thereby reflecting the hierarchical data structure where units at a lower level (participant) belong to only one specific unit at a higher level (simulation group). Time was modeled to compare T1 vs. T2 and to include T3 when applicable. An interaction term between time and professional role was included, adjusted by time since the participant’s most recent Italian Resuscitation Council (IRC) course and, for follow-up analyses, for time elapsed since the simulation session. LMER was chosen as the available literature supports the analysis of Likert scales using parametric models, which have proven to be robust in handling such outcome measures and to account for individuals’ nested random effects [24]. Missing data were handled by excluding from each LMER analysis only those individuals with missing values in the variables included in that specific model.

Statistical significance was set at *p* < 0.05. All analyses were performed using R software (version 4.1.1; R Foundation for Statistical Computing, Vienna, Austria).

Qualitative responses concerning participants’ perceptions of Sim-SEA were analyzed using an inductive qualitative content analysis. Responses expressing multiple concepts were segmented into separate units of meaning and independently coded by two researchers (FM and RB) both of whom were not directly involved in training delivery or debriefing facilitation. FM contributed to the conceptualization and methodological development of the Sim-SEA program, while RB was also involved in statistical analysis. Both researchers were familiar with the overall study objectives and the Sim-SEA framework. Codes were iteratively labelled and grouped into categories through constant comparison. Disagreements were resolved through discussion until consensus was reached.

## 3. Results

### 3.1. Participant Characteristics and Sessions

Of the 64 eligible healthcare professionals invited to participate, 59 took part in the study (overall response rate, RR = 92.2%). The RR was 100% among senior physicians and residents and 79.2% among nurses (19/24). The final sample included 10 senior physicians (16.9%), 30 residents (50.8%), and 19 nurses (32.2%). The five nurses who did not participate were unavailable during the study period because of routine staffing changes. Detailed participant characteristics and baseline self-perceived competence scores are reported in Table 2a,b.

**Figure 2:**
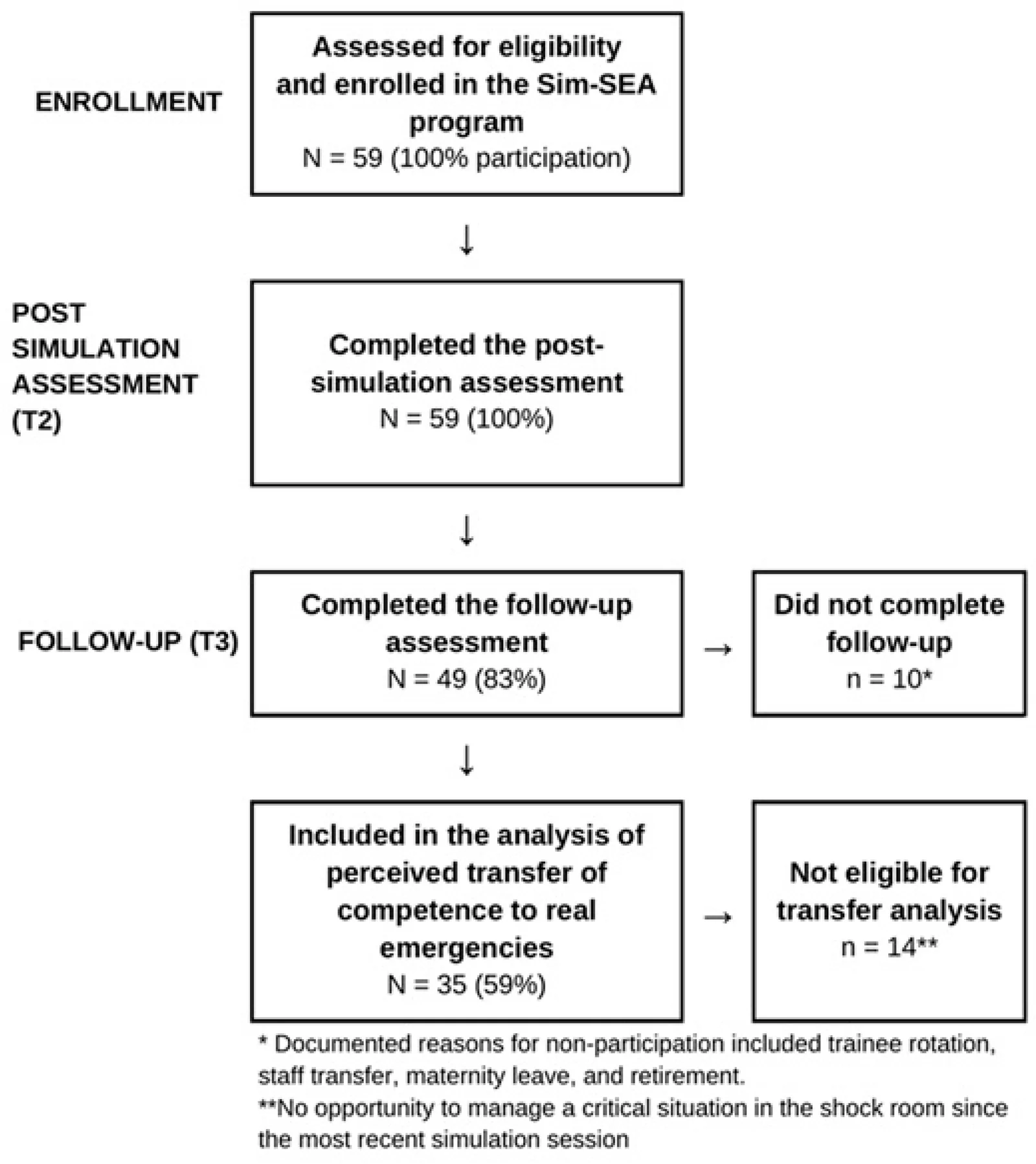
Participant flow diagram.

Ten Sim-SEA cycles were conducted between January and December 2024. All participants completed the T2 post-simulation assessment, whereas 49 (83.1%) completed the T3 follow-up assessment. Loss to follow-up (*n* = 10) was attributable to routine workforce turnover, including maternity leave, staff transfers, and retirement, rather than to withdrawal related to the Sim-SEA intervention. Of the 49 participants who completed the T3 follow-up, 35 had subsequently managed at least one real emergency in the shock room and were therefore included in the analysis of perceived transfer of competence to clinical practice. The participant flow diagram and the numbers of participants included in each analysis are shown in Figure 2.

At baseline, median self-perceived competence scores were 4 (IQR 3–4) for ABCDE assessment, 4 (IQR 2–4) for teamwork, and 3 (IQR 2–4) for situational awareness, communication, speak-up behavior, and decision-making. Baseline scores differed significantly across professional groups for teamwork (*p* < 0.001), situational awareness (*p* < 0.001), speak-up behavior (*p* < 0.001), and decision-making (*p* = 0.021), with residents reporting lower scores.

### 3.2. Pre–Post Evaluation: Perceived Learning and Competence

Model-estimated scores showed significant improvements in self-perceived competence across all assessed domains. Estimated mean scores increased for ABCDE assessment, from 3.6 (95% CI 3.4–3.8) at T1 to 4.1 (95% CI 3.8–4.3) at T2 (*p* = 0.004); teamwork, from 3.3 (95% CI 3.0–3.6) to 4.0 (95% CI 3.7–4.3; *p* = 0.002); situational awareness, from 3.2 (95% CI 3.0–3.5) to 4.0 (95% CI 3.7–4.2; *p* < 0.001); communication, from 3.1 (95% CI 2.8–3.4) to 3.7 (95% CI 3.5–4.0; *p* = 0.002); speak-up behavior, from 3.2 (95% CI 3.0–3.5) to 3.8 (95% CI 3.5–4.1; *p* = 0.003); and decision-making, from 2.9 (95% CI 2.7–3.2) to 3.4 (95% CI 3.2–3.7; *p* = 0.006).

**Figure 3:**
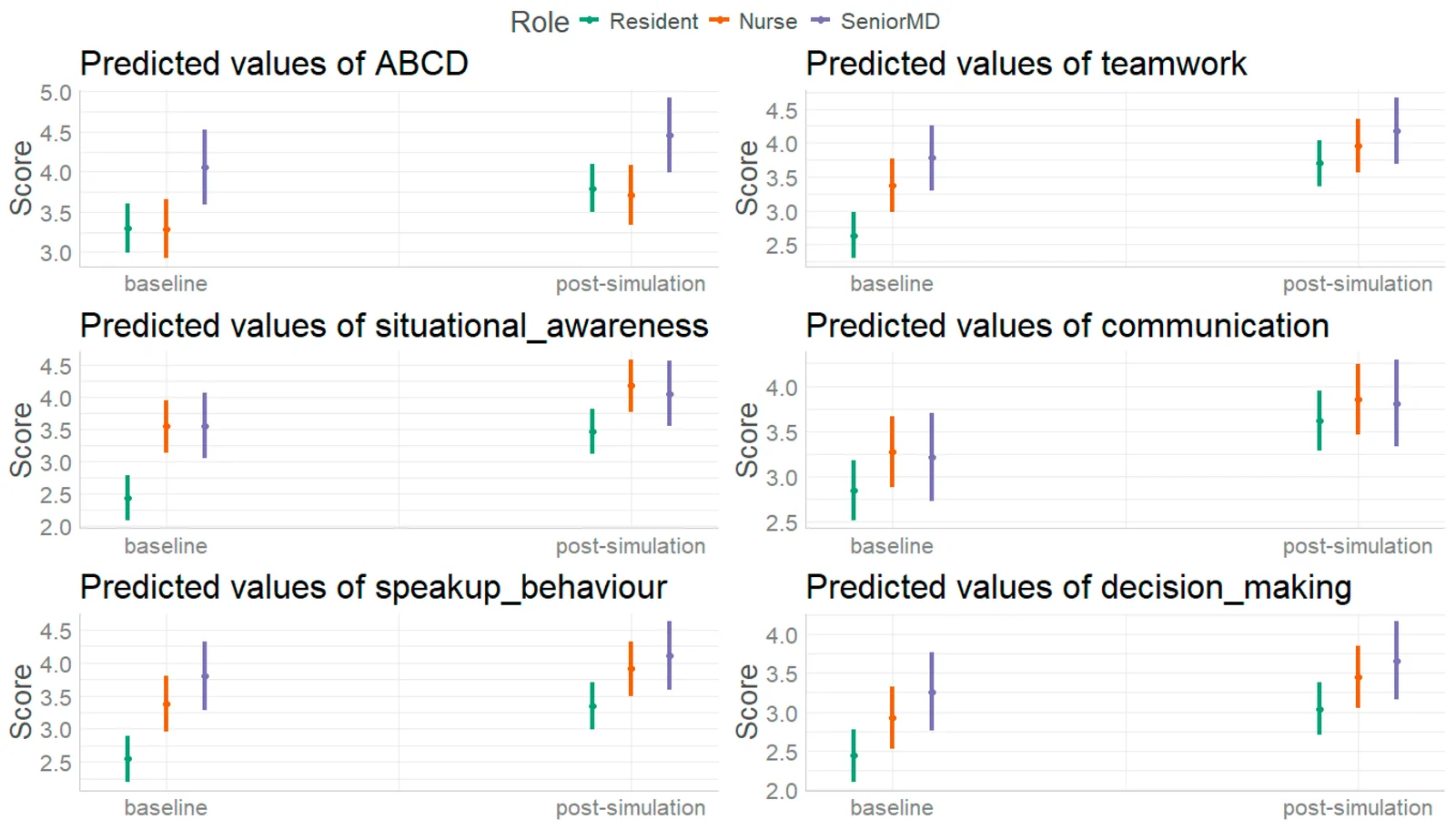
Predicted values of the linear mixed-effect (LMER) models built on the six outcomes as dependent variables and the interaction term between time (T1 = baseline, T2 = post-simulation) and role as independent variables adjusted for time from the last IRC course and with individual by simulation group as a nested random effect.

Residents showed greater improvements than nurses in teamwork (*p* = 0.039) and situational awareness (*p* = 0.040), and greater improvements than senior physicians in the same domains (*p* = 0.024 and *p* = 0.029, respectively). No other significant differences in changes over time were observed across professional roles (Figure 3), and changes were not associated with time since the most recent IRC course.

Overall, 94.9% of participants reported being very or extremely satisfied with the simulation experience.

### 3.3. Organizational and Environmental Changes

Several improvement actions were implemented in the shock room as a result of the Sim-SEA process. Environmental and organizational changes included the relocation of gloves, replacement of the wall clock, reorganization of the whiteboard, and introduction of a drug reference sheet to support accurate dosing during emergencies. The problem identified, action taken, implementation status, and available follow-up evidence are summarized in Supplementary File S5, which also includes illustrative before-and-after photographs of selected environmental changes. Among respondents, the perceived usefulness of these changes was rated as essential or very useful by 28/45 participants (62.2%) for glove relocation (four participants did not respond to this item), 40/49 (81.6%) for wall clock replacement, 44/49 (89.8%) for whiteboard reorganization, and 45/49 (91.8%) for introduction of the drug reference sheet.

In addition, a standard operating procedure (SOP) was developed to address the need for clearer role allocation during shock room management.

### 3.4. Follow-Up: Perceived Transfer to Clinical Practice

**Figure 4:**
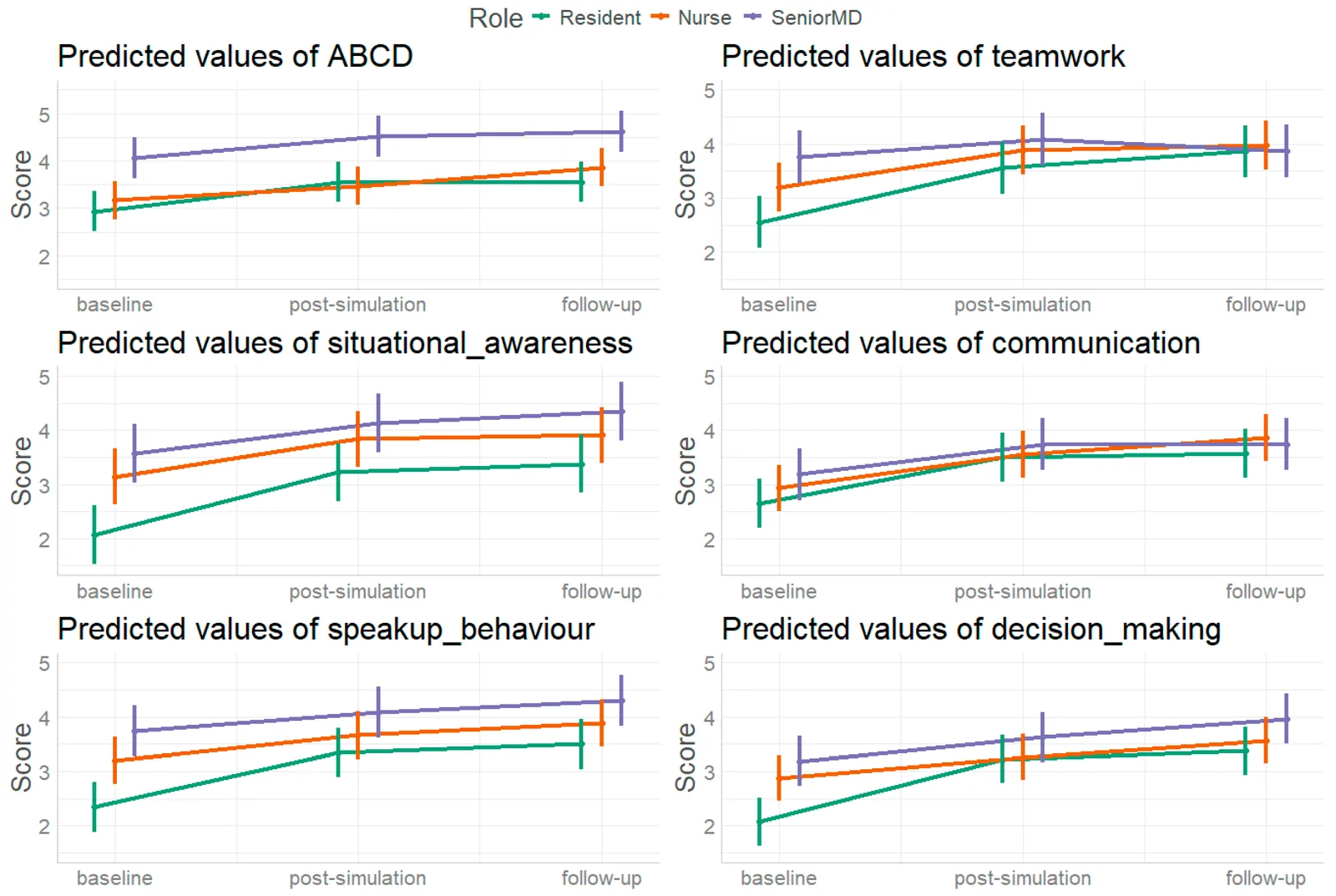
Predicted values of the linear mixed-effect (LMER) models built on the six outcomes as dependent variables and the interaction term between time (T1 = baseline, T2 = post-simulation, T3 = follow-up) and role, as independent variables adjusted for time from the last IRC course and time from the simulation and with individual by simulation group as a nested random effect.

Among the 49 participants assessed at follow-up (T3), 14 had not managed any shock room emergencies since the training and were therefore excluded from the analysis of clinical transfer, leaving 35 participants (59.3% of the initial sample). Among these, 14.3% had completed the Sim-SEA session more than 8 months earlier, 37.1% between 4 and 8 months earlier, and 48.6% less than 4 months earlier. Compared with the 24 participants not included in the transfer-to-practice analysis, the included participants differed in age distribution (median 32 years and IQR, 29.5–44.0 vs. 28 years and IQR, 27–30; *p* = 0.002) and professional-role composition (*p* = 0.021), but not in years of professional experience (*p* = 0.204). Supplementary File S6 provides a detailed descriptive comparison of the two groups. For all six assessed domains, follow-up scores remained significantly higher compared with baseline (T1) (ABCDE *p* = 0.002; teamwork *p* < 0.001; situational awareness *p* < 0.001; communication *p* < 0.001; speak-up behavior *p* < 0.001; decision-making *p* < 0.001), with no significant differences compared with T2. These findings were adjusted for time since simulation and time since the last IRC course (Figure 4).

After managing a real emergency, 82.9% of participants rated the training as extremely useful and 17.1% as very useful. Additionally, 68.6% rated its contribution to stress reduction as very high, 25.7% as high, and 5.7% as moderate.

### 3.5. Sim SEA Acceptability and Perceived Value

Among the 49 respondents, 39 (79.6%) reported being extremely satisfied and 9 (18.4%) very satisfied with the Sim-SEA approach. One participant (2.0%) reported being neither satisfied nor dissatisfied.

All respondents completed the open-ended question exploring their perceptions of Sim-SEA. The responses were segmented into 95 meaning units corresponding to a mean of 1.94 ± 0.97 meaning units per respondent (range: 1–4). The meaning units were grouped into eight categories presented in Table 3 together with representative quotations. Although participants were invited to report both advantages and disadvantages, no negative or less favorable comments were identified.

## 4. Discussion

This pilot study reports preliminary findings on the feasibility and potential value of Sim-SEA as a structured and iterative interface between in situ simulation and clinical risk management within the SEA process. The findings suggest that the implementation of this approach was associated with the identification of environmental and organizational factors contributing to clinical events, improvements in self-perceived competence in teamwork and other non-technical skill domains, collective sensemaking, and the timely translation of improvement priorities into documented organizational changes. These findings align with recent international recommendations, including the Global Consensus Statement on Simulation-Based Practice in Healthcare, which advocates integrating simulation into routine healthcare practice and system-improvement processes to support patient safety, team performance, and workforce resilience [1].

In situ simulation can complement retrospective analysis by allowing teams to examine how clinical work unfolds in practice and to explore interactions among individual performance, team processes, environmental conditions, and system factors that may be only partially accessible through retrospective approaches [25,26,27]. Previous studies have reported associations between in situ simulation embedded within broader system-level initiatives and improved patient outcomes [28].

Participants described Sim-SEA as immersive and highly engaging and reported that reenacting events helped them reconstruct what had occurred, integrate multiple perspectives, and make latent risks, fixation errors, system vulnerabilities, and other implicit aspects of practice more visible. These participant-reported perceptions are in line with previous evidence suggesting that healthcare simulation may support the identification of latent safety threats [29].

Such context-based exploration could contribute to preparedness for infrequent but highly critical situations in pediatric emergency care, where opportunities for experiential learning are limited [27,30,31]. In this sense, Sim-SEA is intended to help teams to learn from clinical practice rather than merely learning about it [12].

From a theoretical perspective, Sim-SEA may be viewed as linking Zones 3 and 4 of the SimZones frameworks, which organizes simulation-based activities according to their learning goals, participant configuration, contextual complexity, and facilitation approach. Zone 3 involves authentic, intact clinical teams working through contextually realistic scenarios and facilitated reflection to explore team behavior, coordination, and system performance. Zone 4 concerns learning and development associated with real patient care and allows actual clinical events to inform subsequent simulation activities [32]. Within this interpretation, Sim-SEA provides a structured and iterative link between the two zones within the SEA process: findings from event analysis inform scenario development; simulation enables teams to re-enter and explore the clinical, relational, environmental, and organizational conditions surrounding the event; and the resulting improvement priorities can be translated into changes in everyday care and revisited in subsequent cycles. Sim-SEA may therefore be understood as providing a bidirectional bridge between Zone 4 and Zone 3 within an established clinical-governance process.

**Figure 5:**
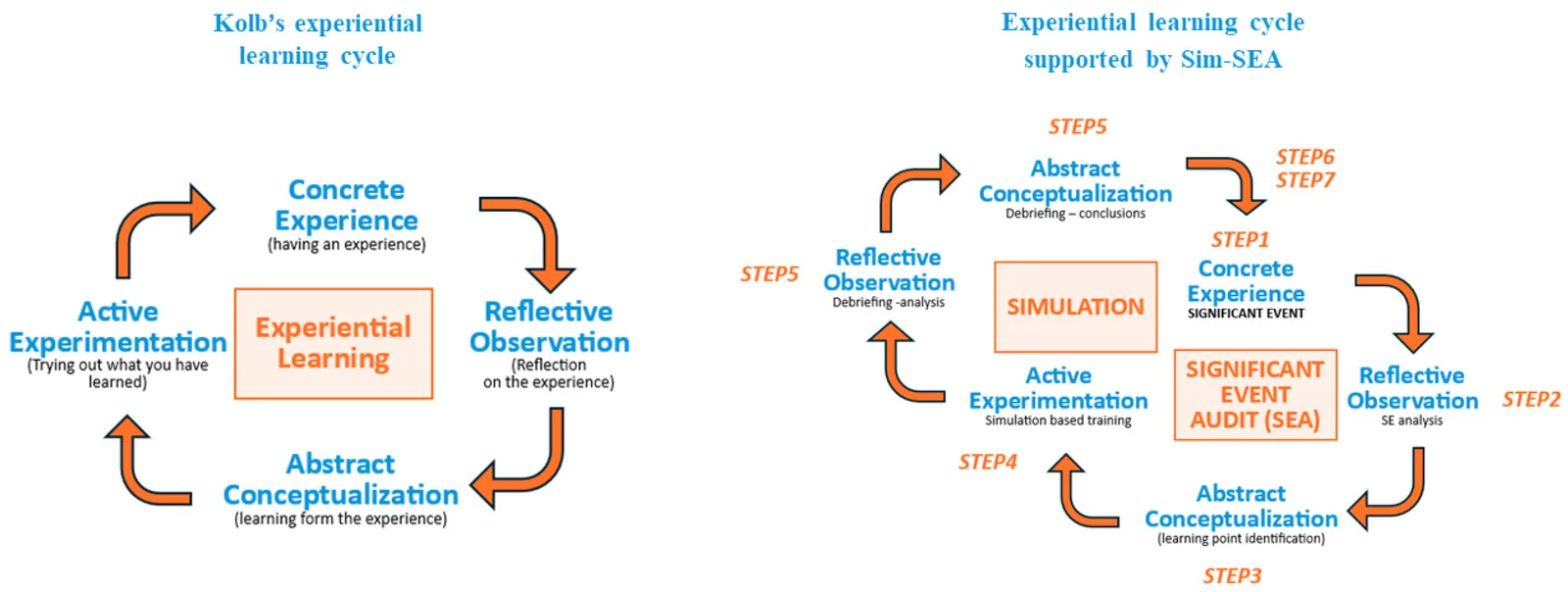
Conceptual Comparison between Kolb’s experiential learning cycle and the expanded experiential learning cycle proposed by Sim-SEA.

The process can also be conceptualized through Kolb’s experiential learning cycle, in which learning develops through the iterative relationship among concrete experience, reflective observation, abstract conceptualization, and active experimentation [33]. Within this conceptualization, the real clinical event represents the initial concrete experience, while structured event analysis supports reflective observation, shared interpretations and the identification of improvement priorities. In situ simulation provides an opportunity to explore and test these insights in a contextually realistic setting, generating a further experience for reflection during debriefing. The resulting learning points and system-improvement actions may then inform subsequent clinical practice and later Sim-SEA cycles, creating the potential for an iterative process of experiential learning, testing, and organizational improvement (Figure 5).

This conceptualization also aligns with the principles of translational simulation, which emphasize the use of simulation to inform improvements in clinical practice and healthcare systems [34]. Closer integration of translational simulation with existing risk-management and quality-improvement processes, including human factors and ergonomics approaches, has been advocated to enhance system-level impact and avoid parallel or fragmented improvement activities [35]. Sim-SEA was designed to support this integration by incorporating event-based in situ simulation as a defined component of the SEA cycle, thereby linking risk identification, experiential exploration, implementation of improvement actions, and subsequent reassessment within a single clinical risk-management process. Its proposed contribution is to provide a formal and repeatable interface between simulation and clinical risk management within a clinical governance framework. In this pilot study, the iterative framework was used to implement and revisit organizational and environmental improvement actions. Changes involving workspace layout, equipment positioning, cognitive aids, and workflows were implemented and subsequently reviewed during later simulation sessions, allowing teams to examine how they functioned in practice and refine them when appropriate. Sim-SEA may therefore offer a structured way to address a recurrent limitation of audit-based improvement processes: translating identified issues into concrete and sustained changes in clinical practice. This is particularly relevant because audit and feedback interventions often have variable and generally modest effects on professional practice [36].

Self-perceived competence scores increased significantly across ABCDE assessment, teamwork, situational awareness, communication, speak-up behavior, and decision-making. These changes are compatible with previous evidence suggesting that simulation may support individual clinical competencies and team performance [37,38].

In exploratory role-stratified analyses, improvements in teamwork and situational awareness were greater among residents than among nurses and senior physicians. Given the small role-specific sample sizes, particularly among senior physicians, these exploratory findings should be interpreted cautiously and may reflect baseline differences or sampling variability rather than differential effects of Sim-SEA. It may nevertheless indicate that in situ simulation may be particularly valuable in supporting residents’ participation and role development within the clinical team. Qualitative accounts similarly reflected participants’ perceptions of improved role clarity, team coordination, and communication, as well as better retention and recall of learning.

Within the Kirkpatrick framework, the immediate increases in self-perceived competence may be considered learner-reported outcomes potentially relevant to Level 2 [39]. Among participants who subsequently managed a real emergency in the shock room, scores across all six domains remained higher than baseline after adjustment for time since simulation and the most recent IRC course and all respondents rated Sim-SEA as very or extremely useful for managing real emergencies. These findings suggest perceived transfer to practice and may be relevant to Level 3, although they do not demonstrate behavioral change because clinical performance was not directly observed. The organizational changes described above represent system-level outputs potentially relevant to Level 4 as they extend beyond individual learning to changes in the clinical environment and work processes; however, their objective effectiveness, sustainability, and effects on patient outcomes were not assessed [40].

Participants also reported lower perceived stress when managing emergencies after taking part in Sim-SEA. Because stress was not objectively measured and no comparison group was available, this finding should be interpreted as a perception rather than as evidence of a demonstrated reduction in stress. In their accounts participants attributed this perception to greater familiarity with critical situations, clearer roles, shared reflection, and a psychologically safe learning environment. Psychological safety—a shared belief that the team is safe for interpersonal risk-taking—is an important condition for team learning, staff well-being, and open communication in healthcare [41,42,43].

Participants perceived the experiential and participatory nature of Sim-SEA as more supportive of open discussion and shared learning than retrospective audit alone. Reported improvements in speak-up behavior, together with accounts of more collaborative interactions, may indicate the potential for Sim-SEA to support less hierarchical team dynamics and a safety culture grounded in learning and collective vigilance [44].

Sim-SEA can also be interpreted through a Safety-II perspective, as it supports learning not only from failures and adverse outcomes but also from the successful adaptations and resources that enable teams to manage complex situations [9]. Participants perceived simulation-based debriefing as helping to make these adaptive processes more visible and actionable than retrospective audit alone. By enabling teams to re-experience and reflect on adaptive responses within their clinical and organizational context, Sim-SEA may help identify resources relevant to resilient team functioning and high-reliability practice, including anticipation of risk, sensitivity to changing conditions, and collective efforts to prevent harm [45]. These potential implications were not directly assessed in the present study.

Finally, the use of in situ simulation may contribute to the feasibility of Sim-SEA, particularly in organizations without dedicated simulation centers. Delivery within the clinical environment can facilitate functional alignment with relevant tasks, workflows, equipment, and contextual conditions without necessarily requiring technologically sophisticated simulation resources. This rationale aligns with contemporary perspectives on simulation fidelity, which emphasize functional task alignment over technological sophistication [46].

Several limitations should be considered. The single-center, observational design and absence of a comparison group limit causal inference and may restrict generalizability, as the observed T1-T2 changes may also reflect concurrent exposure to the introductory CRM session, routine IRC-course activities or other time-related influences rather than the specific contribution of Sim-SEA. The structured nature of the approach may nevertheless support transferability to comparable clinical and organizational contexts. Moreover, most educational and practice-related outcomes were based on self-reported perceptions and ad hoc questionnaires rather than direct observation of clinical behavior or objective clinical and patient-safety outcomes. The self-report instruments were not formally validated in this study, and responses may have been influenced by social-desirability bias. Moreover, in the six-item questionnaire assessing self-perceived competence in selected technical and non-technical domains, each assessed domain was represented by a single item, which limits the evaluation of complex constructs such as teamwork, communication, and decision-making. These questionnaire-based measures should therefore be interpreted as exploratory indicators within the evaluation of the Sim-SEA process, rather than as comprehensive assessments of non-technical skills or actual team performance. Role-specific analyses were based on small subgroup sizes and should therefore be considered exploratory.

Follow-up competence was assessed only among participants who had subsequently managed a real emergency, which may have introduced selection effects. In addition, 17% of participants did not complete the follow-up assessment. Documented reasons for non-participation reflected routine workforce changes, including trainee rotations, staff transfers, maternity leave, and retirement, rather than reported withdrawal related to the intervention. Moreover, because multiple outcomes, comparisons across time points, and exploratory subgroup analyses were examined, the risk of an inflated Type I error rate cannot be excluded. Finally, the variable interval between the most recent simulation and the follow-up assessment may have influenced participants’ self-perceived competence in different ways. Although the follow-up analyses were adjusted for time since the most recent simulation and time since the most recent IRC course, residual confounding related to the timing of the assessment and intervening clinical experiences cannot be excluded.

Despite these limitations, the convergence of quantitative findings, qualitative accounts, and documented organizational changes provides encouraging preliminary support for the feasibility and potential value of Sim-SEA and warrants further evaluation through multicenter studies using comparison groups and objective behavioral, organizational, and clinical outcomes.

## 5. Conclusions

This study introduces Sim-SEA as a structured approach to support the implementation of in situ simulation within an established clinical risk-management process. Its specific contribution may be to provide a formal and reproducible interface between simulation and clinical risk management, with the potential to support the integration into routine clinical-governance processes. By linking event analysis, experiential exploration, implementation of improvement actions, and subsequent reassessment within a single iterative cycle, Sim-SEA may offer a structured opportunity to support team learning, identify system vulnerabilities and adaptive capacities, and translate safety priorities into concrete organizational changes.

These preliminary findings support the feasibility of the approach and suggest participant acceptability and perceived value in a pediatric emergency setting. Further multicenter and comparative studies should incorporate longitudinal designs and objective behavioral, organizational, and clinical outcomes to evaluate its impact, sustainability, and transferability across different healthcare contexts.

## Figures and Tables

**Table 1:** The Sim-SEA phases (adapted from SEA 7 phases) [18].

Phases	Sim-SEA
1. Awareness and Prioritization of a Significant Event	Staff are informed of the opportunity to report significant events—whether adverse, near-miss, no-harm, or positive—to the project lead and the Core Project Team (CPT). The CPT is responsible for prioritizing and selecting events based on their potential for organizational learning and system improvement, as well as their frequency, severity, and relevance to current practice.
2. Information Gathering and Event Reconstruction	The CPT collaborates with staff members involved in the event to reconstruct the sequence of actions, contextual elements, and relevant circumstances surrounding the event.
3. SEA Preliminary Analysis, Key Learning Needs Identification, and Scenario Development	The CPT meets regularly (e.g., monthly) to perform a preliminary analysis of each event using a human factors framework that considers team dynamics, task-related factors, organizational processes, and environmental conditions. Identifies key learning needs at individual and team levels, together with potential system vulnerabilities, protective factors, and priorities for improvement emerging from the analysis of the event and its context. Develops event-based scenarios aligned with these learning and system-improvement priorities and designed to recreate the key clinical, cognitive, relational, environmental, and organizational conditions that shaped performance. Selects appropriate team members to participate in simulation sessions, promoting multidisciplinary engagement, and enabling equitable participation over time.
4. Simulation-Based Investigation and Analysis of the Significant Event	In situ simulation sessions allow staff—including those not involved in the original event—to re-experience key clinical, cognitive, relational, and contextual challenges emerging from the SEA. Structured debriefing using PEARLS supports psychologically safe reflection on team performance, addressing both strengths and areas for improvement, while exploring environmental and organizational contributors—such as workflow, coordination, resource availability, and physical layout. Each session concludes with clearly defined take-home messages, including key learning points, required changes, and positive practices to be reinforced.
5. Agree, Implement, and Monitor Change	Issues identified during the audit–simulation process are translated into targeted actions at individual, team, organizational, or system levels. Subsequent simulation cycles are used to monitor and refine these actions over time.
6. Write It Up	Key insights from each debriefing are documented and shared with staff to support collective learning. Organizational tools (e.g., SOPs, checklists, and workflows) are created or updated accordingly.
7. Report, Share, and Review	Periodic review meetings are held with all staff to share findings, evaluate improvements, and define priorities for future cycles.

**Table 2:** (**a**). Participant characteristics by professional group. (**b**). Baseline self-perceived competence scores by professional role.

(**a**)				
**Characteristics**	**Total (*N* = 59)**	**Senior Physicians (*n* = 10)**	**Residents (*n* = 30)**	**Nurses (*n* = 19)**
**Age, median (IQR)**	30 (28–40)	37 (34–44)	29 (27–30)	41 (29–52)
**Years of pediatric experience, *n* (%)**				
0–2 years	14 (23.7)	0 (0.0)	11 (36.7)	3 (15.8)
3–5 years	22 (37.3)	0 (0.0)	19 (63.3)	3 (15.8)
6–15 years	15 (25.4)	9 (90.0)	0 (0.0)	6 (31.6)
>15 years	8 (13.6)	1 (10.0)	0 (0.0)	7 (36.8)
**Highest-level pediatric IRC course completed, *n* (%)**				
PBLSD	13 (22.0)	0 (0.0)	12 (40.0)	1 (5.3)
EPILS	27 (45.8)	0 (0.0)	13 (43.3)	14 (73.7)
EPALS	19 (32.2)	10 (100.0)	5 (16.7)	4 (21.1)
**Time since most recent pediatric IRC course, *n* (%)**				
<1 year	31 (52.5)	4 (40.0)	17 (56.7)	10 (52.6)
1–2 years	19 (32.2)	2 (20.0)	10 (33.3)	7 (36.8)
3–4 years	7 (11.9)	3 (30.0)	3 (10.0)	1 (5.3)
>4 years	2 (3.4)	1 (10.0)	0 (0.0)	1 (5.3)
(**b**)				
**Baseline Self-Perceived Competence, Median (IQR)**	**Total (*N* = 59)**	**Senior** **Physicians**	**Residents**	**Nurses**
ABCDE assessment	4 (3–4)	4 (4–5)	3.5 (3–4)	4 (3–4)
Teamwork	4 (2–4)	4 (3–4)	3 (2–3)	4 (3–4)
Situational awareness	3 (2–4)	4 (3–4)	2 (2–3)	4 (3–4)
Communication	3 (2–4)	3 (3–4)	3 (2–4)	3 (3–4)
Speak-up behaviour	3 (2–4)	4 (3–4)	2 (2–3)	4 (3–4)
Decision-making	3 (2–4)	3 (3–4)	2 (2–3)	3 (3–4)

Data are presented as median (IQR) or *n* (%). Percentages in the professional-group columns are calculated within each group; percentages in the Total column are calculated using *N* = 59. In Table 2b scores were self-reported on a 5-point Likert scale and represent observed descriptive values. IQR, interquartile range; IRC, Italian Resuscitation Council; PBLSD, Pediatric Basic Life Support and Defibrillation; EPILS, European Pediatric Immediate Life Support; EPALS, European Pediatric Advanced Life Support.

**Table 3:** Categories identified through inductive content analysis, with representative illustrative quotes.

Categories	Description	Examples
Experiential and active engagement	Sim-SEA was described as an immersive and active experience that enabled participants to directly engage with clinical events “step into” the situation, and move beyond purely retrospective approaches, supporting deeper learning.	*Greater direct involvement, not retrospectively* *You truly immerse yourself in the case* *You actively engage within a team, experiencing first-hand the dynamics of the critical event.* *It allows testing of the environment, decision-making processes, and interactions among healthcare professionals*
Realism and fidelity	The value of in situ simulation was strongly linked to its alignment with real clinical, environmental, and organizational conditions, enhancing relevance, credibility, and perceived transferability to practice.	*It enables the recreation of a realistic clinical atmosphere and working environment.* *The more closely the simulation reflects reality, the better one is able to manage a similar event with awareness of its dynamics and environment*
Reflective learning and sensemaking	Sim-SEA supported individual and collective sensemaking by enabling participants to reconstruct and interpret complex clinical events through real-time experience and reflection, integrating multiple perspectives more effectively than retrospective analysis (typically conducted from a more detached, external perspective)	*Being able to discuss the event in real time (rather than retrospectively) but with a clearer and calmer mind to reinterpret it more clearly.* *… a more effective way to focus on and make sense of what had been done* *… by integrating more effectively the perspectives of those directly involved with those of external observers* *It provided insight into how I would have behaved in that specific scenario*
Retention and learning consolidation	Simulation-based experiences were perceived as more memorable and easier to internalize than traditional approaches, supporting retention, recall, and preparedness for future clinical situations.	*Simulated cases are more easily retained and internalized compared to traditional learning approaches* *Errors made during simulation are remembered more vividly …* *Initial perceptions may at times be distorted, and revisiting and correcting them (through simulation and debriefing) helps consolidate and improve the learning achieved through the process*
Improved awareness of risks and errors	Simulation increased awareness of both actual and potential errors, including latent risks and fixation errors, by making cognitive processes and event dynamics more visible during clinical scenarios.	*…… it allows participants to pay closer attention to the sequence of actions and underlying mental processes leading to both actual and potential errors.* *“Living through” a potential adverse event has a greater impact on awareness.* *It allowed direct observation and experiential understanding of the sources of error* *… it helps to recognize and overcome fixation errors.*
Role clarity, teamwork, and communication	Simulation and debriefing supported a clearer understanding of individual roles, team coordination, and communication processes. Participants described improvements in task distribution, coordination, time and resource management, and greater awareness of others’ contributions.	*It enabled greater awareness of each team member’s role within the team.* *It has a direct impact on improving teamwork and communication.* *Improved management of time and resources, and more effective communication among team members*
Psychological safety and emotional regulation	Compared to traditional SEA, Sim-SEA was perceived as providing a psychologically safe environment that enabled open, non-judgmental discussion of errors and experiences, while also supporting participants in actively managing emotional responses—such as stress and anxiety—and building confidence in handling critical situations.	*The most important added value (compared to audit) lies in managing and training the emotional component through immersion in the scenario* *It prepares you emotionally to deal with similar real events much better than just talking about the case afterwards.* *It helps you manage anxiety more effectively* *It allows participants to immerse themselves in errors in a non-judgmental environment*
Positive feedback and learning from success (Safety-II)	Simulation-based debriefing supported recognition and reinforcement of effective behaviors, promotion of positive feedback, and learning from successful performance.	*It also highlights what was done well and experienced positively.* *It provides positive reinforcement for aspects of colleagues’ performance that were appreciated.* *… recognition of what had been done well and less well*

## Data Availability

The data presented in this study are available on request from the corresponding author due to participant confidentiality.

## Supplementary Materials

The following supporting information can be downloaded at https://www.mdpi.com/article/10.3390/healthcare14213810/s1, File S1: SQUIRE 2.0 reporting checklist; File S2: Comparison of the original SEA framework and its Sim-SEA adaptation; File S3: From Significant Event Audit to Simulation Scenario Design: An Illustrative Sim-SEA Application; File S4: Self-perceived competence questionnaire; File S5: Examples of organizational and environmental improvement actions implemented through Sim-SEA; File S6: Comparison of participants included and not included in the perceived transfer-to-practice analysis.

## Abbreviations

The following abbreviations are used in this manuscript:

ABCDE approach	Airway, Breathing, Circulation, Disability, and Exposure
CRM	Crisis Resource Management
CARP	Comitato di Approvazione per la Ricerca sulla Persona (Committee for the Approval of Research Involving Human Participants)
CPT	Core Project Team
IR	Incident reporting
IQR	Interquartile Range
IRC	Italian Resuscitation Council
LMER	Linear Mixed-Effects Model
PER	Pediatric Emergency Room
SOP	Standard operating procedure
SQUIRE	Standards for QUality Improvement Reporting Excellence
PEARLS framework	Promoting Excellence and Reflective Learning in Simulation
SEA	Significant Event Audit
