# Supplementary material for: Sim-SEA: Integrating Significant Event Audit and In Situ Simulation to Support Patient Safety Learning and Organizational Change—A Prospective Single-Center Pilot Study in Pediatric Emergency Care
Source: Healthcare (Basel). 2026 Nov 8;14(21):3810. doi: 10.3390/healthcare14213810 (PMC13762908; doi:10.3390/healthcare14213810)
Supplement: Supplementary file 1 [file healthcare-14-03810-s001.zip › healthcare-4539923-supplementary.pdf]

Supplementary File S1. SQUIRE 2.0 reporting checklist.

	Item Description	Location (or reason for not reporting)
**Title and Abstract**		
1. Title	Indicate that the manuscript concerns an initiative to improve healthcare (broadly defined to include the quality, safety, effectiveness, patient-centredness, timeliness, cost, efficiency and equity of healthcare).	Title: title and study description.
2. Abstract	2a. Provide adequate information to aid in searching and indexing.<br>2b. Summarise all key information from various sections of the text using the abstract format of the intended publication or a structured summary such as: background, local problem, methods, interventions, results, conclusions.	Abstract: Background/Objectives, Methods, Results, Conclusions.
**Introduction**		
3 & 4. Problem description & Available Knowledge	1. Nature and significance of the local problem.<br>2. Summary of what is currently known about the problem, including relevant previous studies.	Introduction, paragraphs 1–4: local patient-safety problem, SEA limitations, and relevant simulation context.
5. Rationale	Informal or formal frameworks, models, concepts, and/or theories used to explain the problem, any reasons or assumptions that were used to develop the intervention(s), and reasons why the intervention(s) was expected to work.	Introduction, paragraphs 5–6: Safety-I/Safety-II rationale and rationale for integrating SEA with in-situ simulation.
6. Specific aims	Purpose of the project and of this report.	Introduction, final paragraphs: primary aims and secondary exploratory aim.
**Methods**		
7. Context	Contextual elements considered important at the outset of introducing the intervention(s).	Methods, Participants and setting; The Sim-SEA approach; Implementation of the Sim-SEA approach: pilot study design and iterative cycles.
8. Intervention(s)	1. Description of the intervention(s) in sufficient detail that others could reproduce it<br>2. Specifics of the team involved in the work.	Methods, The Sim-SEA approach; Implementation of the Sim-SEA approach: pilot study design and iterative cycles; Significant event selection, analysis and scenario development; Debriefing: the PEARLS framework; Table 1; Figure 1; Supplementary Files S2–S3.
9. Study of the Intervention(s)	1. Approach chosen for assessing the impact of the intervention(s)<br>2. Approach used to establish whether the observed outcomes were due to the intervention(s).	Methods, Evaluation and data collection; Statistical analysis; Qualitative analysis; study design described at the beginning of Materials and Methods.
10. Measures	1. Measures chosen for studying processes and outcomes of the intervention(s), including rationale for choosing them, their operational definitions, and their validity and reliability	Methods, Evaluation and data collection; Statistical analysis; Supplementary File S4. Instrument development and limitations of measurement validity are also

	2. Description of the approach to the ongoing assessment of contextual elements that contributed to t…	addressed in Discussion, Limitations.
11. Analysis	1. Qualitative and quantitative methods used to draw inferences from the data<br>2. Methods for understanding variation within the data, including the effects of time as a variable.	Methods, Statistical analysis and Qualitative analysis: descriptive statistics, linear mixed-effects models, handling of time and role interactions, missing data, and qualitative content analysis.
12. Ethical considerations	Ethical aspects of implementing and studying the intervention(s) and how they were addressed, including, but not limited to, formal ethics review and potential conflict(s) of interest.	Methods, beginning of Materials and Methods and Ethical considerations/institutional review statement; end matter, Institutional Review Board Statement, Informed Consent Statement, and Conflicts of Interest.
**Results**		
13 a & b. Evolution of the intervention and details of process measures	13a. Initial steps of the intervention(s) and their evolution over time (e.g., time-line diagram, flow chart, or table), including modifications made to the intervention during the project.<br>13b. Details of the process measures and outcome.	Results, 3.1 Participant characteristics and sessions; 3.2 Pre–post evaluation; 3.3 Organizational and environmental changes; Figure 1; Figure 2; Supplementary File S5.
13 c, d & e Contextual elements and unexpected consequences	13c. Contextual elements that interacted with the interventions<br>13d. Observed associations between outcomes, interventions and relevant contextual factors<br>13e. Unintended consequences such as benefits, harms, unexpected results, problems or failures associated with the intervention(s)	Results, 3.2 Pre–post evaluation; 3.3 Organizational and environmental changes; 3.4 Follow-up: perceived transfer to clinical practice; 3.5 Sim-SEA acceptability and perceived value; Tables 2–3; Figures 2–4; Supplementary Files S5–S6.
13 e. Missing data	13e. Details about missing data.	Results, 3.1 Participant characteristics and sessions and 3.4 Follow-up: perceived transfer to clinical practice; Figure 2; Supplementary File S6.
**Discussion**		
14. Summary	14a. Key findings, including relevance to the rationale and specific aims<br>14b. Particular strengths of the project	Discussion, opening paragraphs: summary of key findings and relevance to the study aims; strengths discussed in relation to the integration of simulation and clinical risk management.
15. Interpretation	15a. Nature of the association between the intervention(s) and the outcomes<br>15b. Comparison of results with findings from other publications<br>15c. Impact of the project on people and systems<br>15d. Reasons for any differences between observed and anticipated outcomes, including the influence of context<br>15e. Costs and strategic trade-offs, including opportunity costs	Discussion, paragraphs addressing interpretation, comparison with previous literature, impact on people and systems, contextual influences, and organizational change. No formal cost or opportunity-cost analysis was undertaken.
16. Limitations	16a. Limits to the generalisability of the work.<br>16b. Factors that might have limited internal validity such as confounding, bias, or imprecision in the design, methods, measurement, or analysis.	Discussion, Limitations: generalisability, causal inference, lack of comparison group, self-reported/ad hoc measures,

	16c. Efforts made to minimise and adjust for limitations.	subgroup size, missing follow-up data, timing, confounding, and Type I error.
17. Conclusion	17a. Usefulness of the work<br>17b. Sustainability<br>17c. Potential for spread to other contexts<br>17d. Implications for practice and for further study in the field<br>17e. Suggested next steps	Discussion, final paragraph and Conclusions: usefulness, transferability, implications for practice and future research. Sustainability and spread were not formally evaluated.
**Other information**		
18. Funding	Sources of funding that supported this work. Role, if any, of the funding organisation in the design, implementation, interpretation and reporting.	End matter, Funding: no external funding was received; no role of a funding organisation was applicable.

Supplementary File S2. Comparison of the original SEA framework and its Sim-SEA adaptation

Table S1. SEA description and Sim-SEA adaptation

Original SEA phase	Core purpose in the original SEA	Corresponding Sim-SEA phase	Adaptation introduced in Sim-SEA
**1. Awareness and prioritization of a significant event**	Staff identify and priorities relevant events for collective review and routine learning.	**1. Awareness and Prioritization of a Significant Event**	Largely retained, with event selection formalized through the Core Project Team (CPT) and prioritized according to its potential for organizational learning and system improvement.
**2. Information gathering**	Factual information is collected from staff accounts, written records, clinical documentation, and other relevant sources to reconstruct the event.	**2. Information Gathering and Event Reconstruction**	Information gathering is focused on reconstructing the event trajectory and the clinical, contextual, and organizational circumstances needed to inform simulation-based exploration.
**3. The facilitated team-based meeting**	A facilitated, non-blaming team discussion supports event analysis, identification of learning needs, and agreement on actions.	**3. SEA Preliminary Analysis, Key Learning Needs Identification, and Scenario Development**	The facilitated discussion becomes a preliminary SEA-informed analysis aimed at identifying learning needs, system vulnerabilities, protective factors, adaptive behaviors, and scenario-design elements.
**4. Analysis of the significant event**	The event is analyzed by asking what happened, why it happened, what was learned, and what has been changed or actioned.	**4. Simulation-Based Investigation and Analysis of the Significant Event**	Analysis is extended through in situ simulation and structured debriefing, allowing teams to re-experience key challenges and explore team performance, contextual dynamics, and system contributors.
**5. Agree, implement, and monitor change**	Agreed actions are implemented and monitored over time to assess whether changes reduce the likelihood of recurrence.	**5. Agree, Implement, and Monitor Change**	Improvement actions are implemented and can be reassessed, monitored, and refined through subsequent simulation cycles conducted in the clinical context.
**6. Write it up**	A comprehensive anonymized record documents the event analysis, learning, and agreed or implemented actions.	**6. Write It Up**	Documentation includes debriefing insights, learning points, agreed actions, and revised organizational tools such as SOPs, checklists, or workflows.
**7. Report, share, and review**	Learning is reported and shared through governance channels so that others can benefit from the analysis.	**7. Report, Share, and Review**	Findings are shared with staff and governance structures; periodic review also informs priorities for future Sim-SEA cycles.

Note. SEA = Significant Event Audit; CPT = Core Project Team; SOPs = standard operating procedures.

Supplementary File S3. From Significant Event Audit to Simulation Scenario Design: An Illustrative Sim-SEA Application

This appendix provides an illustrative example of how a real clinical event was systematically transformed into a structured in-situ simulation scenario within the Sim-SEA framework, informed by both a Safety-I perspective (analysis of what went wrong) and a Safety-II perspective (analysis of adaptive and successful performance).

Phase 1. Awareness and prioritization of a significant event

The event selected for this Sim-SEA cycle was initially reported through the incident reporting system (Figure S1) and was considered for inclusion following consultation with the staff involved. The event was subsequently reviewed and prioritized by the Core Project Team (CPT).

It involved a child presenting anaphylaxis who initially received 0.3 mL of diluted intramuscular adrenaline instead of the intended undiluted solution resulting in no immediate clinical improvement and delaying effective treatment. The error was promptly recognized and corrected, and no permanent harm occurred.

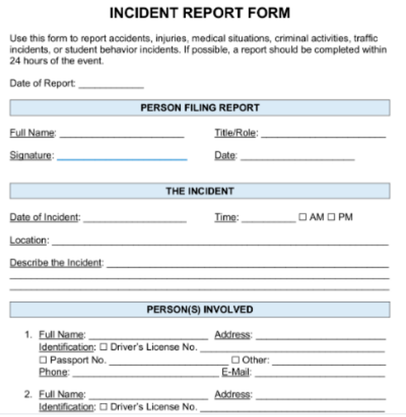
INCIDENT REPORT FORM

Use this form to report accidents, injuries, medical situations, criminal activities, traffic incidents, or student behavior incidents. If possible, a report should be completed within 24 hours of the event.

Date of Report: ___________

PERSON FILING REPORT

Full Name: ___________ Title/Role: ___________

Signature: ___________ Date: ___________

THE INCIDENT

Date of Incident: ___________ Time: ________ ☐ AM ☐ PM

Location: ___________

Describe the Incident: ___________

PERSON(S) INVOLVED

1. Full Name: ___________ Address: ___________
   Identification: ☐ Driver's License No. ___________
   ☐ Passport No. ___________ ☐ Other: ___________
   Phone: ___________ E-Mail: ___________
2. Full Name: ___________ Address: ___________
   Identification: ☐ Driver's License No. ___________

Figure S1 The incident report form

During the prioritization phase, CPT classified the event as a no-harm event and selected it based on its high learning potential. Selection criteria included the time-critical nature of pediatric anaphylaxis, the involvement of interprofessional communication and medication preparation under pressure, and the relevance of the event to routine clinical practice and existing protocols.

Phase 2. Information gathering and event reconstruction

2.1 Information gathering

Information was collected by the Core Project Team (CPT) in collaboration with the healthcare professionals directly involved in the event. Data sources included:

- the Emergency Department medical record,
- whiteboards used during real-time clinical case management,
- an interdisciplinary debriefing conducted shortly after the event.

This process enabled the reconstruction of a shared timeline, which served as the basis for identifying contextual, cognitive, and organizational contributing factors.

2.2 Timeline of the Significant Event

- A 5-year-old child developed urticaria, abdominal pain, and shortness of breath after ingestion of a walnut-containing cake.
- The correct triage code was assigned.
- Anaphylaxis was promptly and correctly diagnosed.
- The physician verbally prescribed 0.3 ml of intramuscular adrenaline, without specifying that it should be administered undiluted.
- The nurse administered 0.3 ml of diluted adrenaline, resulting in no immediate clinical improvement.
- A second dose of adrenaline, at the correct concentration, was administered and led to rapid clinical improvement.

Phase 3. SEA preliminary analysis, key learning needs identification and scenario development

3.1 The identification of contributing factors

The analysis followed the "Five Whys" approach, exploring both why things went wrong (Figure S2) and why no harm occurred (Figure S3).

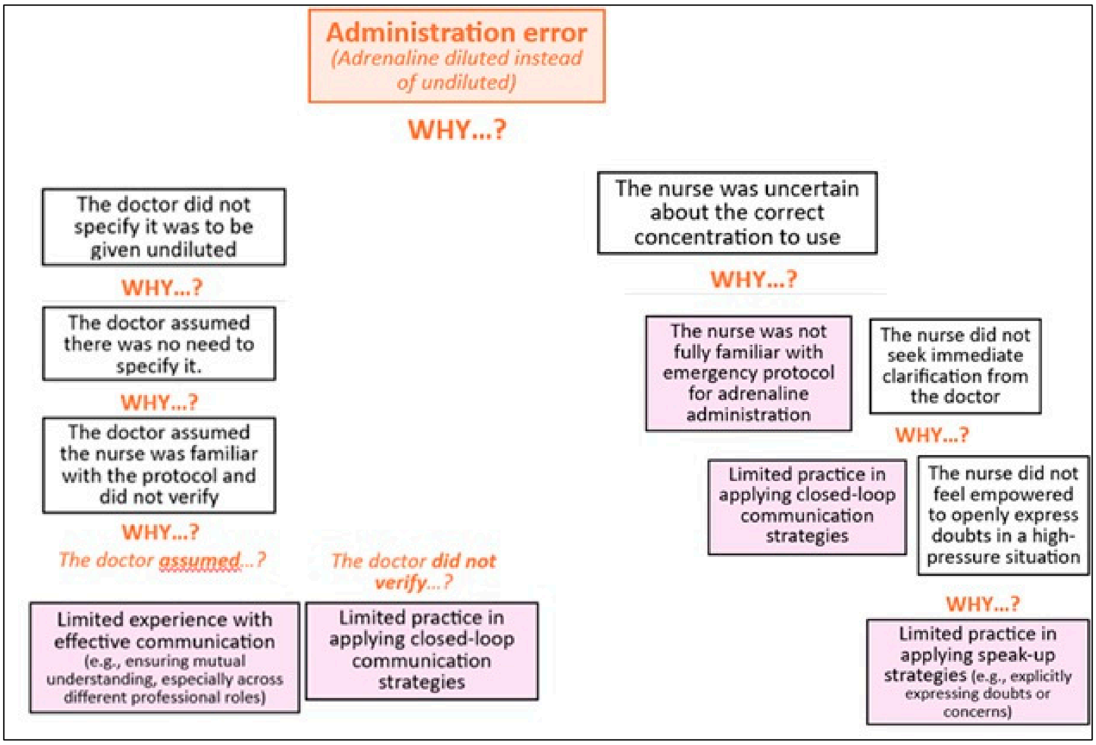

Figure S2. Application of the Five Whys method to explore why things went wrong in a no-harm medication event and to identify contributing factors (highlighted in red).

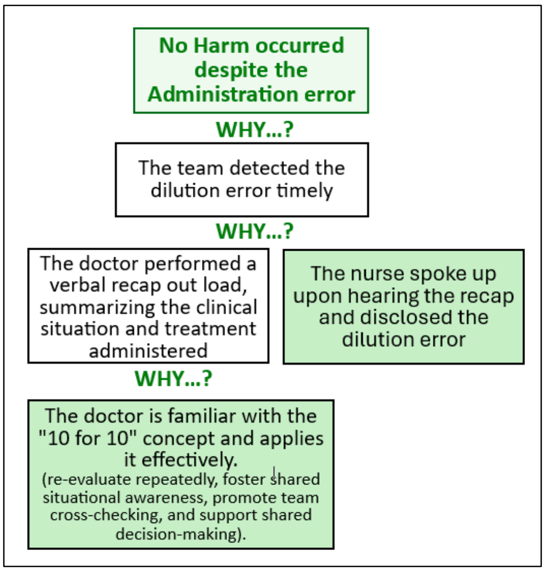

Figure S3. Application of the Five Whys method to explore why things went well in a no-harm medication event and to identify protective and resilience factors (highlighted in green).

This dual analysis allowed the identification of both system weaknesses and existing protective factors.

3.2 Identification of Key Learning Needs

Based on the preliminary SEA analysis, the CPT identified a set of learning needs derived from both the analysis of what went wrong and the analysis of what went well. Behaviors and practices that contributed to

vulnerabilities were targeted for focused training, while Effective behaviors supporting safe performance were identified for reinforcement and dissemination to support individual and team resilience. Together, these learning points informed the design of the simulation scenario.

Areas for improvement included:

- Familiarity with adrenaline administration protocols in pediatric anaphylaxis.
- Clear and explicit communication across different professional roles.
- Systematic use of closed-loop communication.
- Encouraging explicit expressions of doubts and speaking up.

Positive aspects to reinforce were:

- Shared situational awareness.
- Periodic team "time-outs" (e.g., "10 seconds for 10 minutes").
- Prompt reassessment and adaptive response.

3.3 Translation into Scenario Design

The identified learning points were translated into an event-based in situ simulation scenario designed to recreate the clinical, cognitive, relational, environmental, and organizational conditions surrounding the real event within a controlled simulation setting.

The following table illustrates the progression from SEA findings to learning and system-improvement priorities and corresponding scenario elements.

Table S2. From findings to scenario elements

Five Whys finding	Learning or system-improvement priority	Scenario elements
**Limited experience with effective communication**, particularly in ensuring mutual understanding across different professional roles.	Clear and explicit communication across professional roles.	A multidisciplinary pediatric emergency team with clearly defined roles. The physician provides a verbal prescription for adrenaline, requiring explicit communication of the concentration and intended administration.
**Limited practice in applying closed-loop communication strategies.**	Systematic use of closed-loop communication.	A critical verbal instruction requiring the nurse to repeat back the prescription, confirm the adrenaline concentration, and obtain clarification before administration. The physician is required to verify shared understanding.
**Limited familiarity with the emergency protocol for adrenaline administration.**	Familiarity with adrenaline administration protocols in pediatric anaphylaxis.	Medication preparation and administration during a time-critical anaphylaxis scenario, including recognition of the correct adrenaline concentration, dose, and route.
**The nurse did not feel empowered to openly express doubts in a high-pressure situation.**	Encouraging explicit expression of doubts and speaking up.	A high-pressure clinical situation in which the nurse is uncertain about the adrenaline concentration and must decide whether and how to express concern to the physician and the team.
**Limited practice in speaking-up strategies**, including explicitly expressing doubts or concerns.	Encouraging and reinforcing speaking-up behaviours across professional roles.	The scenario requires the nurse to raise a concern about the medication preparation or administration. The team leader's response to the concern becomes part of the team interaction explored during debriefing.

Five Whys finding	Learning or system-improvement priority	Scenario elements
**Protective factor: the nurse spoke up after hearing the verbal recap and disclosed the dilution error.**	Reinforcement of speaking up, shared situational awareness, and team cross-checking.	A verbal team recap provides an opportunity for the nurse to disclose the medication error, allowing the team to identify the discrepancy and intervene before harm occurs.
**Protective factor: the physician was familiar with and effectively applied the "10 seconds for 10 minutes" concept**, supporting repeated reassessment, shared situational awareness, cross-checking, and shared decision-making.	Reinforcement of shared situational awareness, periodic team time-outs, and prompt reassessment and adaptive response.	Structured team time-outs and repeated ABCDE reassessment are incorporated into the scenario. The patient's clinical evolution requires the team to reassess the response to treatment, recognize persistent instability, correct the medication error, administer the appropriate treatment, and adapt the management plan.

An ad hoc scenario design sheet was developed to structure the simulation, including patient information, baseline clinical parameters, expected progression, critical decision points, and predefined evolution pathways depending on participants' actions (Figure S4).

The scenario reproduced realistic environmental conditions, role distribution, and time pressure, allowing participants to experience and reflect on the same critical decision points identified during the real event analysis. Particular attention was paid to replicating real-world workflow, equipment availability, communication patterns, and organizational constraints in order to facilitate the identification of latent system factors and modifiable environmental contributors.

<table>
<tr><td colspan="2">INFORMATIONS</td><td>Normal values for VP</td></tr>
<tr><td colspan="2">Patient arrives accompanied by mother and triage nurse.</td><td>Kg 20
RR 20 apm
HR 110-120 bpm
BP 100 mmHg (min 80 mmHg)</td></tr>
<tr><td rowspan="2">BASELINE</td><td colspan="2">QUICK LOOK (B, B, B): stridor widespread urticaria, very agitated</td></tr>
<tr><td>Assessment
A: pervious

B: polypnea, chest recessions and distress, wheezing SpO2 87% RR 25/min

C: HR 170/min, CRT 4 sec, central pulses ok, weak peripheral pulses, BP 80/40 mmHg, no preload

D: A AVPU, agitated

Glycemia 115 mmHg

E: T 36,3°C, widespread urticaria</td><td>Parameters

SpO2 87% in air, RR 30/min

HR 170, BP 80/40 mmHg

T 36,3</td></tr>
<tr><td>CORRECT ACTIONS</td><td colspan="2">- O2 with reservoir mask
- IM adrenaline
- vascular access
- blood test (tryptase)
- iv steroid
- 2nd dose of Adrenaline</td></tr>
<tr><td>EVOLUTION</td><td colspan="2">1. If O2 → RR 30 apm SpO2 95/96%.
2. Adrenaline within 5 min -> persistent signs of shock
2.1 if administration 2nd dose of adrenaline within 5 mins ->improvement
2.2 if not administration 2nd dose of adrenaline within 5 min by 1st→ V/P of AVPU, HR 100 BPM, BP 60/50, worsening of rash
3. If not adrenaline within 5 minutes → V/P of AVPU, HR 100 BPM, BP 60/50 mmHg, worsening of rash
4. if not adrenaline within 8 minutes- cardiac arrest with asystole</td></tr>
</table>

Figure S4. Structured template used to translate learning points from the Significant Event Audit into an event-based in-situ simulation scenario

Supplementary File S4. Self-perceived competence questionnaire

Please indicate how confident you feel in your own ability to perform each of the following tasks during the management of an emergency patient in the shock room.

Please select one response for each question using the following 5-point scale:

1 = not at all confident; 2 = slightly confident; 3 = moderately confident; 4 = very confident; 5 = extremely confident.

The behaviours listed in the third column are examples to consider when answering the corresponding question and should not be rated separately.

N.	Question	Behaviours to consider when answering	1	2	3	4	5
1	How confident do you feel in using the ABCDE approach?	—	☐	☐	☐	☐	☐
2	How confident do you feel in working as part of a team during the management of a patient in the shock room?	*Distributing roles; allocating the workload; making shared goals explicit*	☐	☐	☐	☐	☐
3	How confident do you feel in maintaining situational awareness during the management of a patient in the shock room?	*Recognizing the clinical situation and its evolution; using cognitive aids; ensuring the availability of relevant equipment and personnel*	☐	☐	☐	☐	☐
4	How confident do you feel in managing communication in the shock room?	*Communicating all relevant information clearly and explicitly; using an appropriate tone of voice; using closed-loop communication*	☐	☐	☐	☐	☐
5	How confident do you feel in contributing during emergency situations managed in the shock room?	*Promptly requesting help; expressing doubts or seeking clarification; providing frequent updates*	☐	☐	☐	☐	☐
6	How confident do you feel in making decisions during emergency cases in the shock room?	*Avoiding fixation errors; gathering and comparing information; using cross-checks*	☐	☐	☐	☐	☐

Supplementary File S5. Examples of Organizational and Environmental Improvement Actions Implemented through Sim-SEA

This supplementary appendix summarizes the organizational and environmental changes implemented following the Sim-SEA cycles and arising from system-level contributory factors identified during debriefing. Supplementary Table S3 reports, for each change, the problem identified, the action taken, its implementation status, and the available follow-up evidence. Illustrative before-and-after photographs are provided for the changes for which visual documentation was available.

Table S3. Summary of organizational and environmental changes implemented following the Sim-SEA cycles, including the contributory problem identified, the action taken, implementation status, and available follow-up evidence.

**Problem identified**	**Action taken**	**Implementation status**	**Follow-up/evidence**
Limited visibility of elapsed time during emergency management	Replacement of the analogue wall clock with a digital display in the shock room	Implemented	Before-and-after photographs; perceived usefulness rated as essential or very useful by 81.6% of respondents; no formal objective evaluation of effectiveness
Gloves were not readily accessible near the shock-room entrance	Relocation of gloves to a more accessible position near the entrance	Implemented	Before-and-after photographs; perceived usefulness rated as essential or very useful by 62.2% of respondents; no formal objective evaluation of effectiveness
Lack of a standardized visual structure to support documentation of the initial ABCDE assessment and communication of key findings within the team during emergency management	Redesign of the shock-room whiteboard using an EPALS-based ABCDE structure; retention of a secondary board for time tracking and drug administration	Implemented	Before-and-after photographs and photograph of the implemented whiteboard; perceived usefulness of whiteboard reorganisation rated as essential or very useful by 89.8% of respondents; no formal objective evaluation of effectiveness
Need for rapid access to drug-dosing information during emergencies	Introduction of a drug reference sheet to support accurate dosing	Implemented	Participant feedback; perceived usefulness rated as essential or very useful by 91.8% of respondents; no formal objective evaluation of effectiveness
Need for clearer role allocation during shock-room management	Development of a standard operating procedure (SOP) defining roles and responsibilities during shock-room management	Developed	no formal evaluation of its effect on role allocation or team coordination

Example 1. Environmental redesign to support time management

Replacement of the wall clock in the shock room with a digital display to facilitate time tracking during emergency management and support team coordination and situational awareness.

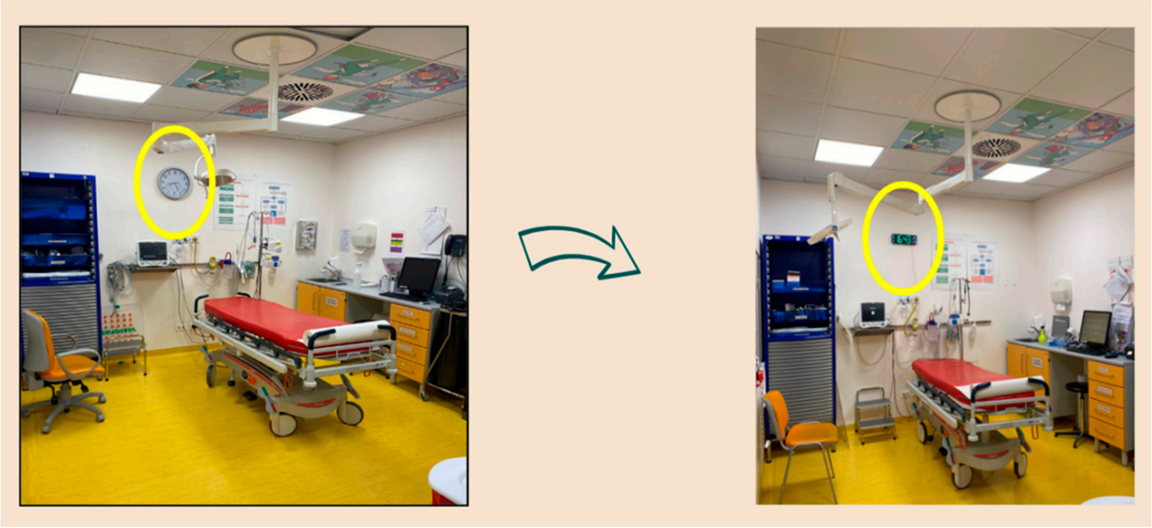

Figure S5. Replacement of the wall clock in the shock room

Example 2. Optimization of PPE accessibility

Relocation of gloves near the shock room entrance to improve accessibility and PPE organization and reduce potential delays in emergency response.

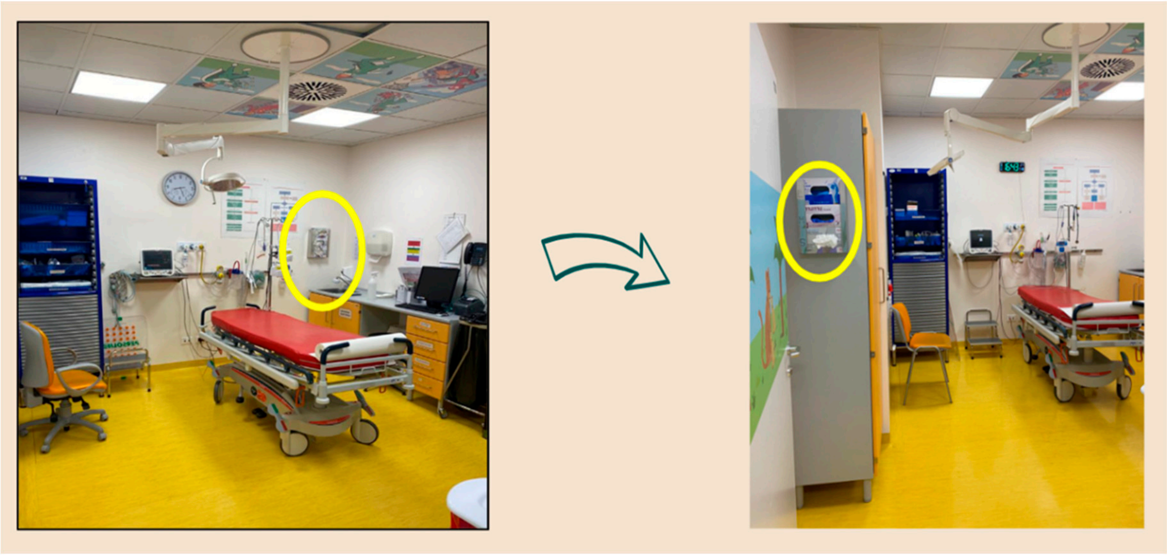

Figure S6. Relocation of gloves near the shock room entrance

Example 3. Standardization of communication and documentation tools

Redesign of the shock-room whiteboard using an EPALS-based ABCDE structure to standardize documentation during the primary survey. A secondary board was retained for time tracking and drug administration to support information sharing and team coordination.

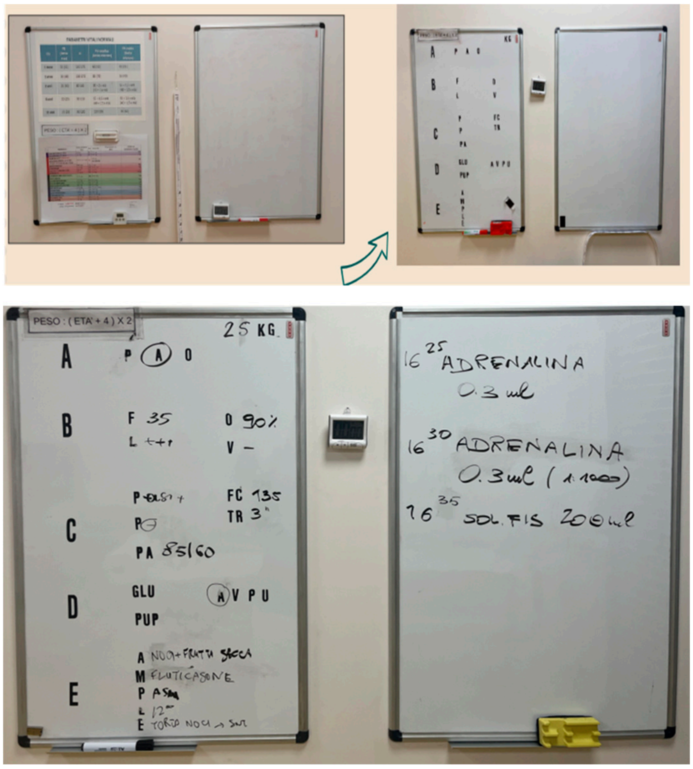

Figure S7. Redesign of the shock-room whiteboard

These examples illustrate how Sim-SEA supports the translation of reflective learning into concrete system-level improvements.

Supplementary File S6. Comparison of participants included and not included in the perceived transfer-to-practice analysis.

Table S4. Characteristics of participants included and not included

Characteristic	Included in transfer analysis (n=35)	Not included (n=24)	p-value
**Age, median (IQR), years**	32 (29.5–44.0)	28 (27–30)	**0.002**
Age range, years	26–57	25–59	—
**Professional role**			**0.021**
Senior physicians, n (%)	9 (25.7)	1 (4.2)	
Residents, n (%)	13 (37.1)	17 (70.8)	
Nurses, n (%)	13 (37.1)	6 (25.0)	
**Professional experience**			**0.204**
0–2 years, n (%)	8 (22.9)	7 (29.2)	
3–5 years, n (%)	9 (25.7)	10 (41.7)	
6–15 years, n (%)	13 (37.1)	3 (12.5)	
>15 years, n (%)	5 (14.3)	4 (16.7)	

Age was compared using the two-sided Mann–Whitney U test. Professional role and years of professional experience were compared using the chi-square test. Comparisons were exploratory and descriptive.
